# Supplementary material for: Altered conformational sampling along an evolutionary trajectory changes the catalytic activity of an enzyme
Source: Nat Commun. 2020 Nov 23;11:5945. doi: 10.1038/s41467-020-19695-9 (PMC7683729; doi:10.1038/s41467-020-19695-9)
Supplement: Supplementary file 1 — Supplementary Information [file 41467_2020_19695_MOESM1_ESM.pdf]

Supplementary Information

Altered conformational sampling along an evolutionary trajectory changes the catalytic activity of an enzyme

Joe A. Kaczmarski[1,7], Mithun C. Mahawaththa[1,7], Akiva Feintuch[2,7], Ben E. Clifton[1,6], Luke A. Adams[3], Daniella Goldfarb[2,8,*], Gottfried Otting[1,4,8,*] & Colin J. Jackson[1,4,5,8,*]

[1] Research School of Chemistry, The Australian National University, Canberra, ACT 2601, Australia.

[2] Department of Chemical and Biological Physics, Weizmann Institute of Science, Rehovot 76100, Israel.

[3] Medicinal Chemistry, Monash Institute of Pharmaceutical Sciences, Monash University, Parkville, VIC 3052, Australia. (Australian Research Council Training Centre for Fragment Based Design and Monash Fragment Platform)

[4] Australian Research Council Centre of Excellence for Innovations in Peptide and Protein Science.

[5] Australian Research Council Centre of Excellence in Synthetic Biology.

[6] Present address: Protein Engineering and Evolution Unit, Okinawa Institute of Science and Technology, 1919-1 Tancha, Onna-son, Okinawa 904-0412, Japan.

[7]These authors contributed equally: Joe A. Kaczmarski, Mithun C. Mahawaththa, Akiva Feintuch

[8]These authors jointly supervised this work: Daniella Goldfarb, Gottfried Otting, Colin J. Jackson

[*]Corresponding authors: daniella.goldfarb@weizmann.ac.il ; gottfried.otting@anu.edu.au ; colin.jackson@anu.edu.au

Supplementary Table 1. Data collection and refinement statistics for the AncCDT-5 X-ray crystal structure.

	**AncCDT-5 (HEPES)**
**PDB**	6WUP
**Data collection**	
Space group	P 6_5 2 2
Cell dimensions	
a, *b*, *c* (Å)	70.95, 70.95, 175.39
α, β, γ (°)	90, 90, 120
Resolution (Å)	35.69–1.49 (1.52–1.49)*
R_{merge}	0.083 (3.91)
R_{pim}	0.014 (0.729)
$I/\sigma I$	22.4 (1.40)
$CC_{1/2}$	1.00 (0.501)
Completeness (%)	100.0 (100.0)
Redundancy	35.3 (30.3)
**Refinement**	
Resolution (Å)	43.85–1.49 (1.52–1.49)
No. reflections	43560 (4265)
R_{work} / R_{free}	0.1975 (0.2972) / 0.2217 (0.3129)
No. atoms	2194
Protein	1882
Ligand/ion	16
Water	296
B-factors	32.5
Protein	31.0
Ligand/ion	28.9
Water	42.1
R.m.s. deviations	
Bond lengths (Å)	0.007
Bond angles (°)	0.89

* Statistics for the highest-resolution shell are shown in parentheses.

Supplementary Table 2. Cα–Cα distances (tag positions) and calculated Gd(III)–Gd(III) distances for crystal structures and representative MD snapshots.[a]

Name of Crystal Structure/MD Snapshot	Cα–Cα distance (nm)	Calculated Gd(III)–Gd(III) distance (nm)
**L-Arg AncCDT-1 (PDB 5T0W)**	**2.61**	**2.94**
MD_Anc1_closed_01	2.52	2.89
MD_Anc1_closed_02	2.63	2.88
MD_Anc1_closed_03	2.73	3.28
MD_Anc1_closed_04	2.82	3.18
MD_Anc1_closed_05	2.91	3.39
**apo-AncCDT-1 (PDB 5TUJ)**	**3.36**	**4.06**
MD_Anc1_open_01	3.19	3.87
MD_Anc1_open_02	3.40	4.90
MD_Anc1_open_03	3.53	4.19
MD_Anc1_open_04*	3.63	4.10
MD_Anc1_open_05	3.65	4.30
MD_Anc1_wide_01	3.79	4.53
MD_Anc1_wide_02	3.95	5.23
MD_Anc1_wide_03	3.97	4.68
MD_Anc1_wide_04	4.07	5.20
MD_Anc1_wide_05	4.17	4.99
**AncCDT-3/L188 (PDB 5JOS)**	**4.21**	**5.25**
MD_Anc3_L188_wide_01	4.04	5.03
MD_Anc3_L188_wide_02	4.07	5.42
MD_Anc3_L188_wide_03	4.26	5.54
MD_Anc3_L188_wide_04	4.46	5.85
MD_Anc3_L188_wide_05	4.67	6.24
MD_Anc3_P188_wide_01	4.10	5.27
MD_Anc3_P188_wide_02	4.27	5.57
MD_Anc3_P188_wide_03	4.56	5.73
MD_Anc3_P188_wide_04	4.68	6.22
MD_Anc3_P188_wide_05	4.78	6.34

Name of Crystal Structure/MD Snapshot	Cα–Cα distance (nm)	Calculated Gd(III)–Gd(III) distance (nm)
**HEPES-AncCDT-5 (PDB 6WUP)**	**2.77**	**3.09**
MD_Anc5_closed_01	2.50	2.79
MD_Anc5_closed_02	2.56	3.09
MD_Anc5_closed_03	2.63	3.11
MD_Anc5_closed_04	2.71	3.42
MD_Anc5_closed_05	2.79	3.41
MD_Anc5_open_01	3.01	3.40
MD_Anc5_open_02	3.11	3.67
MD_Anc5_open_03	3.23	4.00
MD_Anc5_open_04	3.34	4.18
MD_Anc5_open_05	3.44	4.17
MD_Anc5_wide_01	3.56	4.54
MD_Anc5_wide_02	3.68	4.52
MD_Anc5_wide_03	3.93	5.12
MD_Anc5_wide_04	4.06	5.16
MD_Anc5_wide_05	4.21	5.44
**HEPES-*Pa*CDT (PDB 3KBR)**	**2.53**	**3.47**
**Acetate-*Pa*CDT (PDB 5HPQ)**	**2.49**	**3.45**
MD_PaCDT_closed_01	2.58	3.49
MD_PaCDT_closed_02	2.67	3.70
MD_PaCDT_closed_03	2.76	3.84
MD_PaCDT_closed_04	2.82	3.54
MD_PaCDT_closed_05	2.96	3.85
MD_PaCDT_open_01	3.15	3.99
MD_PaCDT_open_02	3.33	4.06
MD_PaCDT_open_03	3.59	4.40
MD_PaCDT_open_04	3.61	4.46
MDPaCDT_open_05	3.81	4.40
**apo-*Tm*ArgBP (PDB 4PRS)**	**4.00**	

[a] Gd(III)–Gd(III) distances were calculated following modelling of the AzF-propargyl-DO3A-Gd(III) residue at each mutation site on structures obtained from crystallography (bold) or MD simulations. The models were built by identifying the most favourable values of the dihedral angles χ_1 and χ_2 of the tag (using the mutation tool of PyMOL (The PyMOL Molecular Graphics System, Version 2.0 Schrödinger, LCC.) and setting χ_6 to 180°[1]. For the MD snapshots, structures with

varying Cα–Cα distances (“MD_...._01-05”) were selected as representative structures from each conformational state (i.e. closed, open, wide-open). The data points representing these structures are shown as colored crosses in Figure 4.

*This snapshot was initially grouped as being open (based on the Cα–Cα distance), but was allocated into the wide-open group based on PCA analysis and radius of gyration measurements.

Supplementary Table 3. Sites mutated for the incorporation of AzF residues.

Protein	Mutation sites[a]
**AncCDT-1**	Q68/K138; Q68/Q219; K138/S161
**AncCDT-3**	Q68/R138
**AncCDT-5**	Q68/R138
***Pa*CDT**[b]	A68/A139
***Se*LAOBP**	A61/T151

[a] Amber stop codons were introduced to incorporate pairs of AzF residues. The positions in the amino acid sequence are indicated together with the original amino acid types.

[b] The mutant A68/R138 was also prepared, but the tagging yields obtained were too low for DEER experiments.

Supplementary Table 4. Primers used in this study.

Primer Name	Sequence
AncCDT-1_Q68F	CCGGCACTGTAGACCGGTAAATTTGATATC
AncCDT-1_Q68R	CAAATTTACCGGTCTACAGTGCCGGAATAATAC
AncCDT-1_K138F	GGCAGCATAGGAATTTCTGCCG
AncCDT-1_K138R	AGAAATTCCTATGCTGCCTGTTCGCTG
AncCDT-1_Q219F	TGGCTGAAATAGATGAAAAAAGACGGC
AncCDT-1_Q219R	GCCGTCTTTTTTCATCTATTTCAGCCAATTATTC
AncCDT-1_S161F	GAGGTTGTTTAGGGTCGTGCAGATGCAATG
AncCDT-1_S161R	CACGACCCTAAACAACCTCTTGAAAG
AncCDT-3_Q68F	GCGATCTGTAGGCAGATAAATTTGATATCGC
AncCDT-3_Q68R	TATCTGCCTACAGATCGCTCATCAGGGTCG
AncCDT-3_R138F	GAAAAATTTGCATAGGAACATCTGAAAAAAGCCAAAATCACCG
AncCDT-3_R138R	GGCTTTTTTCAGATGTTCCTATGCAAATTTTTCATTGGTGCC
AncCDT-5_Q68F	GGATGATTTTTAGGCAGATAAATTTGATATCGCC
AncCDT-5_Q68R	ATCTGCCTAAAAATCATCCATCAGGGTCGG
AncCDT-5_R138F	CGTTTTGCATAGGCCCATCTGAAACAGGCACAG
AncCDT-5_R138R	CAGATGGGCCTATGCAAAACGTTCATTGGTGC
*Pa*CDT_A68F	GCGTGATTTTTAGGATGATCGTTTTGATATTGCCATGAG
*Pa*CDT_A68R	ACGATCATCCTAAAAATCACGCATCAGATTCGGCCAG
*Pa*CDT_A139F	GCACGTTAGAATCTGAAAAAAGCACGCATTCTGG
*Pa*CDT_A139R	TCAGATTCTAACGTGCAAATTTTTCATTGGTGCC
*Se*LAOBP_A61F	CTGAAATAGAAAAAAATCGATGCAATTATTAGCAGC
*Se*LAOBP_A61FR	TTTTTTTCTATTTCAGGCTCGGAATCAGTGC
*Se*LAOBP_T151F	GATCTGTAGGCAGGTCGTCTGGATGCAGCACTGCAG
*Se*LAOBP_T151R	CTGCCTACAGATCGCTATAGATCAGATCCTGATTTGC
AncCDT-1 F	GTTTAACTTTAAGAAGGAGATATACATATG ATCGCAGCAAGCACCCTG
AncCDT-1 R	GCCCTGAAAATACAGGTTTTCGAGTTTAAACCATTTTTCGTACAGTTTGTCATAGG
AncCDT-3 F	ATGGAAAACCTGTATTTTCAGGGCATCGCAGCAAGCCGTCTG
AncCDT-3 R	TACCCGGGAGCTCGAATTCTTATTAGAGTTTCAGCCATTTCTCGTACAGTTTTTC
AncCDT-5 F	ATGGAAAACCTGTATTTTCAGGGCATCGCAGCAAGCCGTCTG
AncCDT-5 R	TACCCGGGAGCTCGAATTCTTAttaGAGTTTCAGCCATTTATCAAAAATACGCTG
*Pa*CDT F	ATGGAAAACCTGTATTTTCAGGGCATCCAAGAAAGCCGTCTGG
*Pa*CDT R	TACCCGGGAGCTCGAATTCTTATTAGAGTTTACCATGTGCGGTCG
*Se*LAOBP F	ACCTGTATTTTCAGGGCGCACTGCCGCAGACCGTTCG
*Se*LAOBP R	CCCGGGAGCTCGAATTCTTAATCGCCATACACGTTAAAGTCGAAGTATTTTTC
pETMCSIII F	GAATTCGAGCTCCCGGGTAC
pETMCSIII R	GCCCTGAAAATACAGGTTTTCCATATG

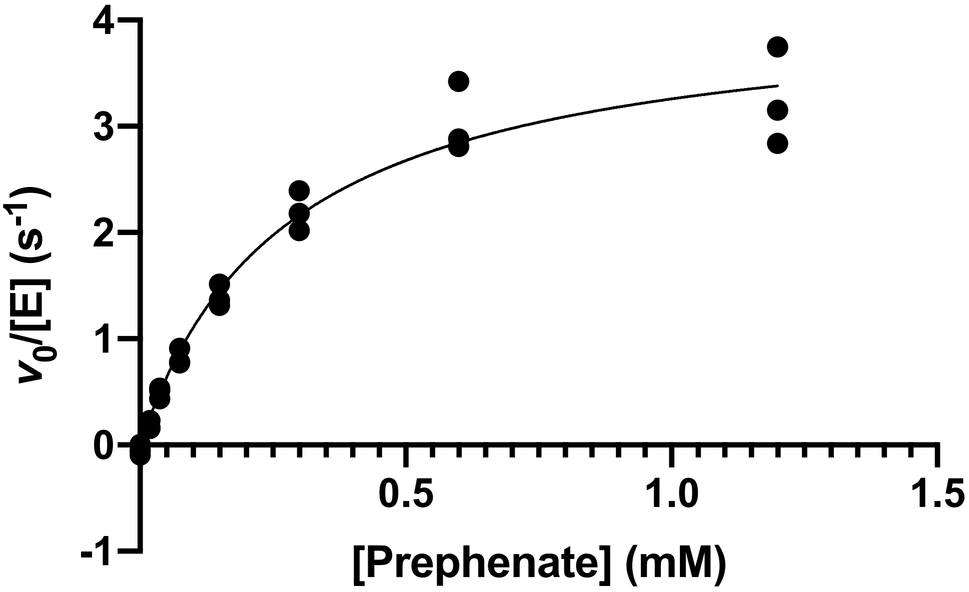

Supplementary Figure 1. Michaelis-Menten plot for AncCDT-5 prephenate dehydratase activity. Data points from three technical replicates are shown (n = 3). Source data are provided as a Source Data file.

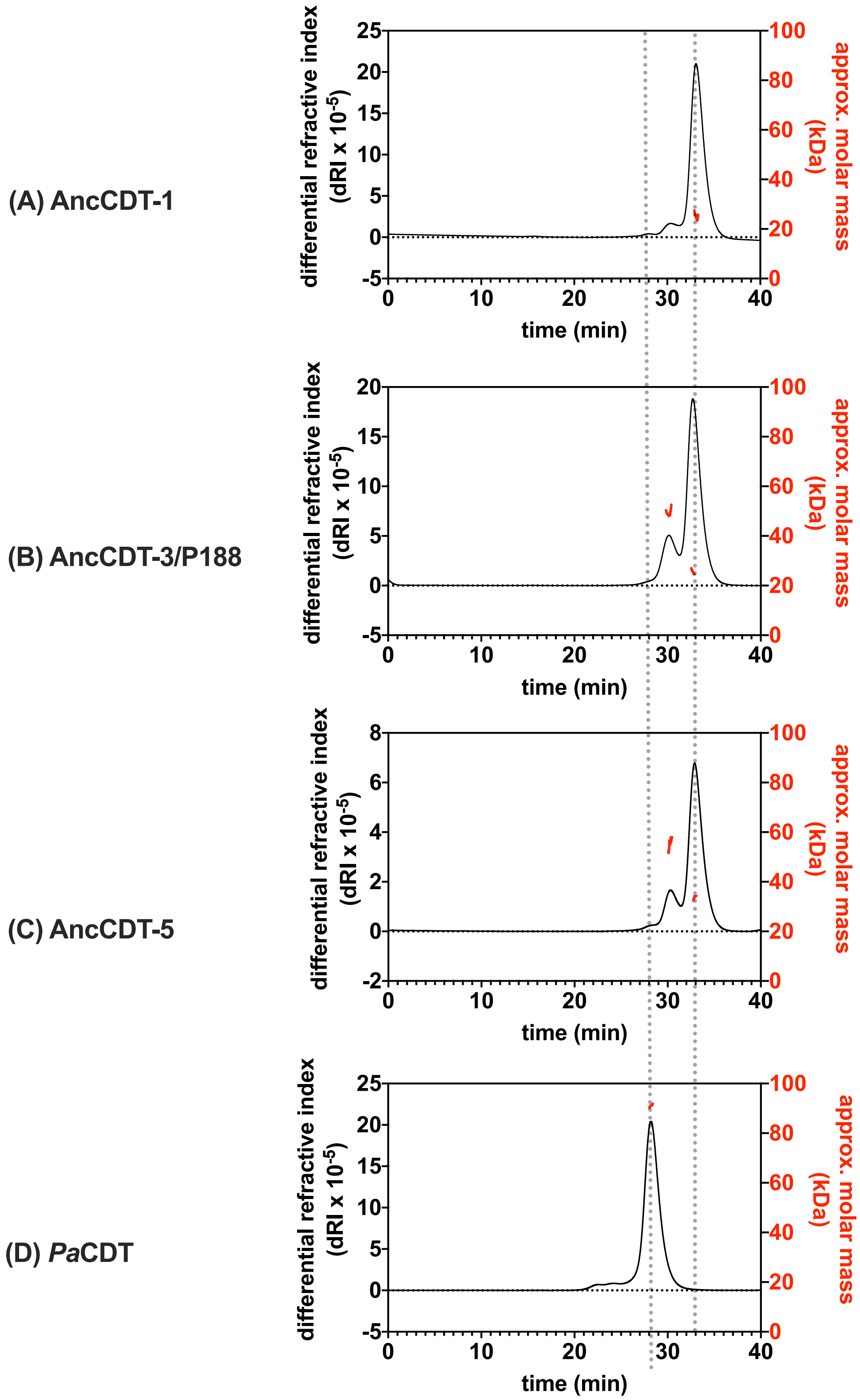

Supplementary Figure 2. SEC-MALS of proteins. Results from analytical size-exclusion chromatography coupled with multiangle light scattering (SEC-MALS) for (A) AncCDT-1, (B) AncCDT-3/P188, (C) AncCDT-5 and (D) *Pa*CDT.

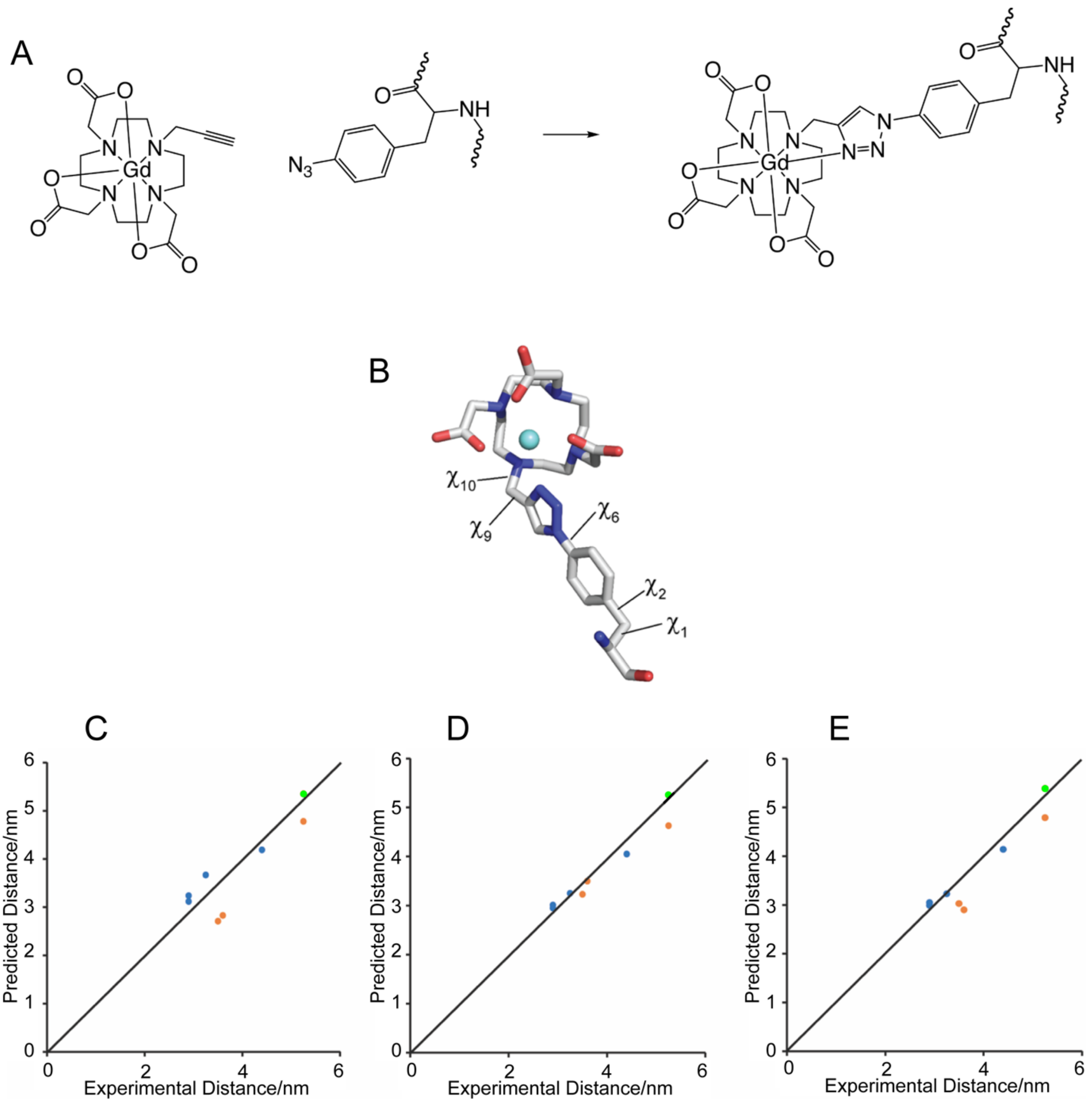

Supplementary Figure 3. The AzF-propargyl-DO3A-Gd(III) residue and correlations between modelled and experimental Gd(III)–Gd(III) distances. (A) Scheme of the Cu(I)-catalyzed cycloaddition reaction used to attach the propargyl-DO3A-Gd(III) tag to the AzF residue[4]. (B) Dihedral angles in the AzF-propargyl-DO3A-Gd(III) residue. $\chi_6 = 0°$ in the conformation shown. (C) Correlation between modelled and experimental Gd(III)–Gd(III) distances, using the crystal structures of AncCDT-1 (blue; PDB ID 5T0W: mutants 68/138, 68/219, 138/161; PDB ID 5TUJ: mutant 68/138), AncCDT-3/P188 (green; PDB ID 5JOS: mutant 68/138) and *Pa*CDT (orange; PDB ID 3KBR: single mutants 68 and 139, and double mutant 68/139). The distances correspond to the maxima of the respective experimental or modelled DEER distance distributions. Distances were predicted using $\chi_6 = 0°$. The angles χ_9 and χ_{10} were

fixed to -140° and 70°, respectively, to allow coordination of the metal ion (cyan sphere in B) by the nearest nitrogen of the triazole ring. χ_1 and χ_2 angles were selected by using those conformations, which were identified by the mutation tool of the program PyMOL to generate minimal steric clashes. (D) Same as (C), but using χ_6 = 180°. This conformation was used to represent the modelled distances in all other figures of the present work. (E) Same as (D), but χ_9 was allowed to be random while the χ_{10} angle was randomly set to -60°, 60°, or 180° with equal likelihood. Although the variation of these dihedral angles broadened the modelled distance distributions, the maxima of the Gd(III)–Gd(III) distance distributions did not change by more than 0.7 nm.

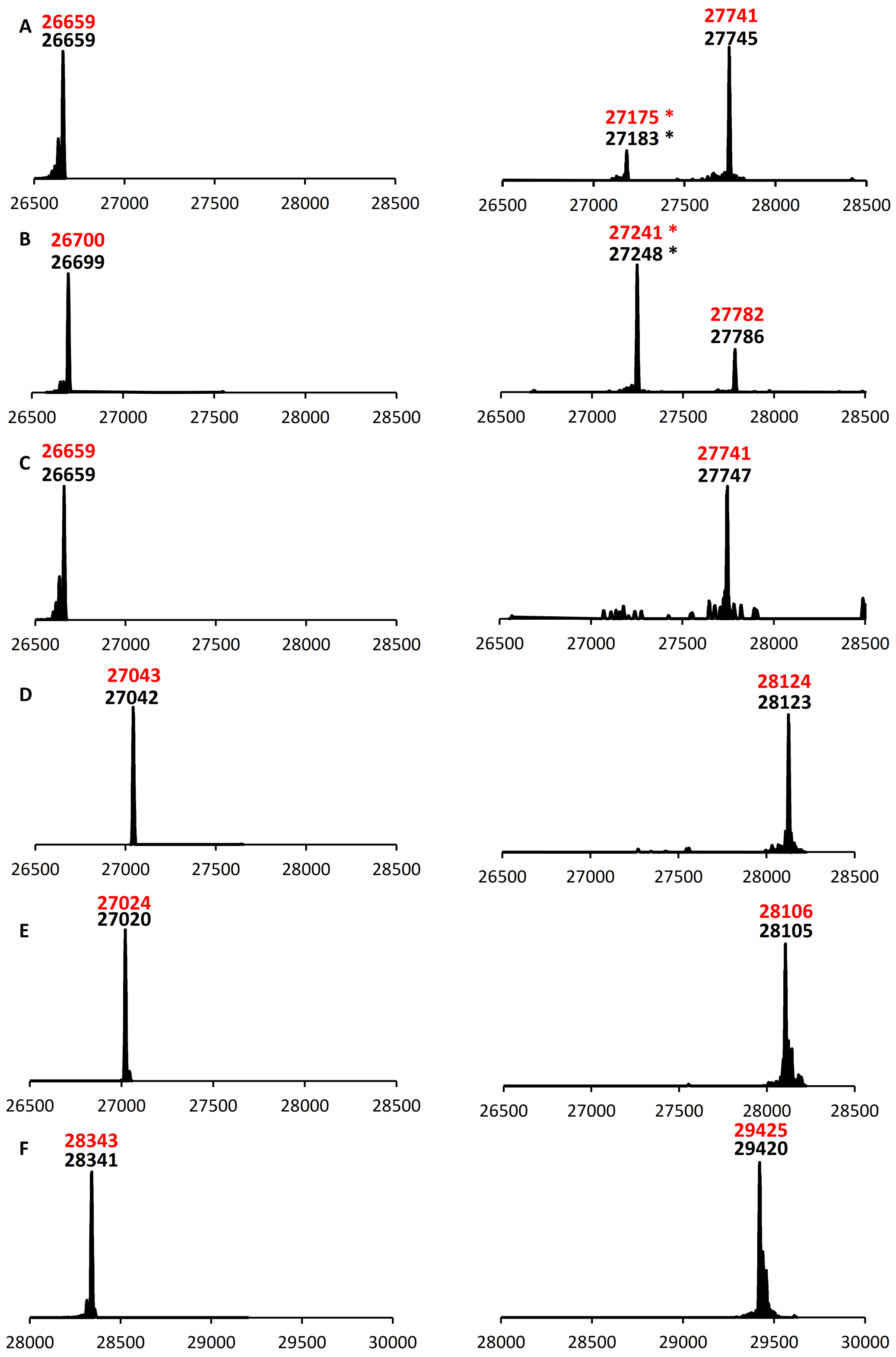

Supplementary Figure 4. Mass spectra of protein samples before and after tagging. The left and right panels show the mass spectra before and after tagging, respectively, together with the expected masses (red). Stars identify mass peaks attributed to singly tagged protein. (A) Refolded AncCDT-1, AzF in positions 68 and 138. (B) Refolded AncCDT-1, AzF in positions 138 and 161.

(C) AncCDT-1, AzF in positions 68 and 219. (D) AncCDT-3, AzF in positions 68 and 138. (E) AncCDT-5, AzF in positions 68 and 138. (F) *Pa*CDT, AzF in positions 68 and 139.

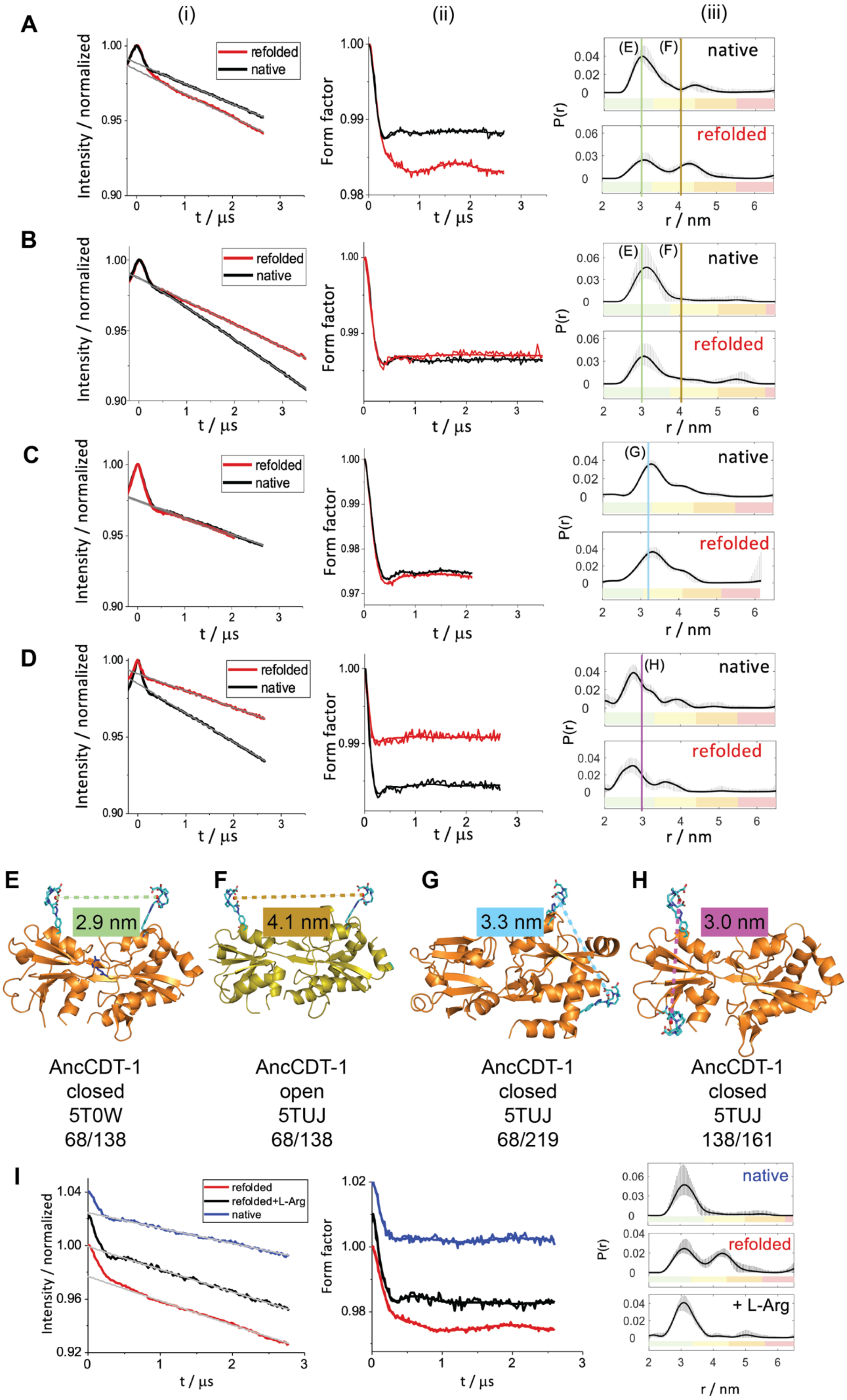
A
B
C
D
E
F
G
H
I
(i)
(ii)
(iii)
refolded
native
refolded+L-Arg
+ L-Arg
Intensity / normalized
Form factor
P(r)
t / µs
r / nm
2.9 nm
4.1 nm
3.3 nm
3.0 nm
AncCDT-1
closed
5T0W
68/138
AncCDT-1
open
5TUJ
68/138
AncCDT-1
closed
5TUJ
68/219
AncCDT-1
closed
5TUJ
138/161

Supplementary Figure 5. Additional and primary DEER data for AncCDT-1. Data from DEER measurements on natively purified (black) and refolded (red) AncCDT-1 samples (100 µM protein in D_2O and 20 % w/v deuterated glycerol) tagged at positions (A) 68 and 138 in the absence of exogenous L-Arg, (B) 68 and 138 in the presence of 1.5-fold molar excess L-Arg, (C) 68 and 219, or (D) 138 and 161. The left panel (i) shows the primary DEER data for the sample and background (gray) decay function. The central panel (ii) shows the DEER form factors after background correction, with fits corresponding to the distance distributions shown in (iii). The right panel (iii) shows the corresponding distance distributions with color coding of the reliability regions as defined in DeerAnalysis, corresponding to the DEER evolution time used (Pale green: the shape of the distance distribution is reliable. Pale yellow: the mean distance and distribution width are reliable. Pale orange: the mean distance is reliable. Pale red: long-range distance contributions may be detectable, but cannot be quantified.). The solid lines represent the distributions with the best r.m.s.d. from the experimental data and the striped regions represent the variation of alternative distributions (± 2 times the standard deviation) obtained by varying the parameters of the background correction and nosie as calculated by the validation tool in the DeerAnalysis software package[5]. The parameter ranges used for the validations were: white noise 0–1.5, background start $0.2*t_{max}$–$0.6*t_{max}$, and background dimension 3–3.6. A limited variation in background dimension was allowed, as a non-homogeneous background improved the fit for some of the traces. Vertical lines represent the predicted Gd(III)–Gd(III) distances when AzF-propargyl-DO3A-Gd(III) residues are modelled on crystal structures of (E) closed AncCDT-1 (PDB 5T0W) at positions 68 and 138, (F) open AncCDT-1 (PDB 5TUJ) at positions 68 and 138, (G) closed AncCDT-1 at positions 68 and 219, or (H) closed AncCDT-1 at positions 138 and 161. (I) Data from a replicate experiment, in which DEER measurements were made on native (blue), refolded (red) and refolded + L-Arg (black) samples of AncCDT-1 (tagged at positions 68 and 138) – as described for (A) and (B).

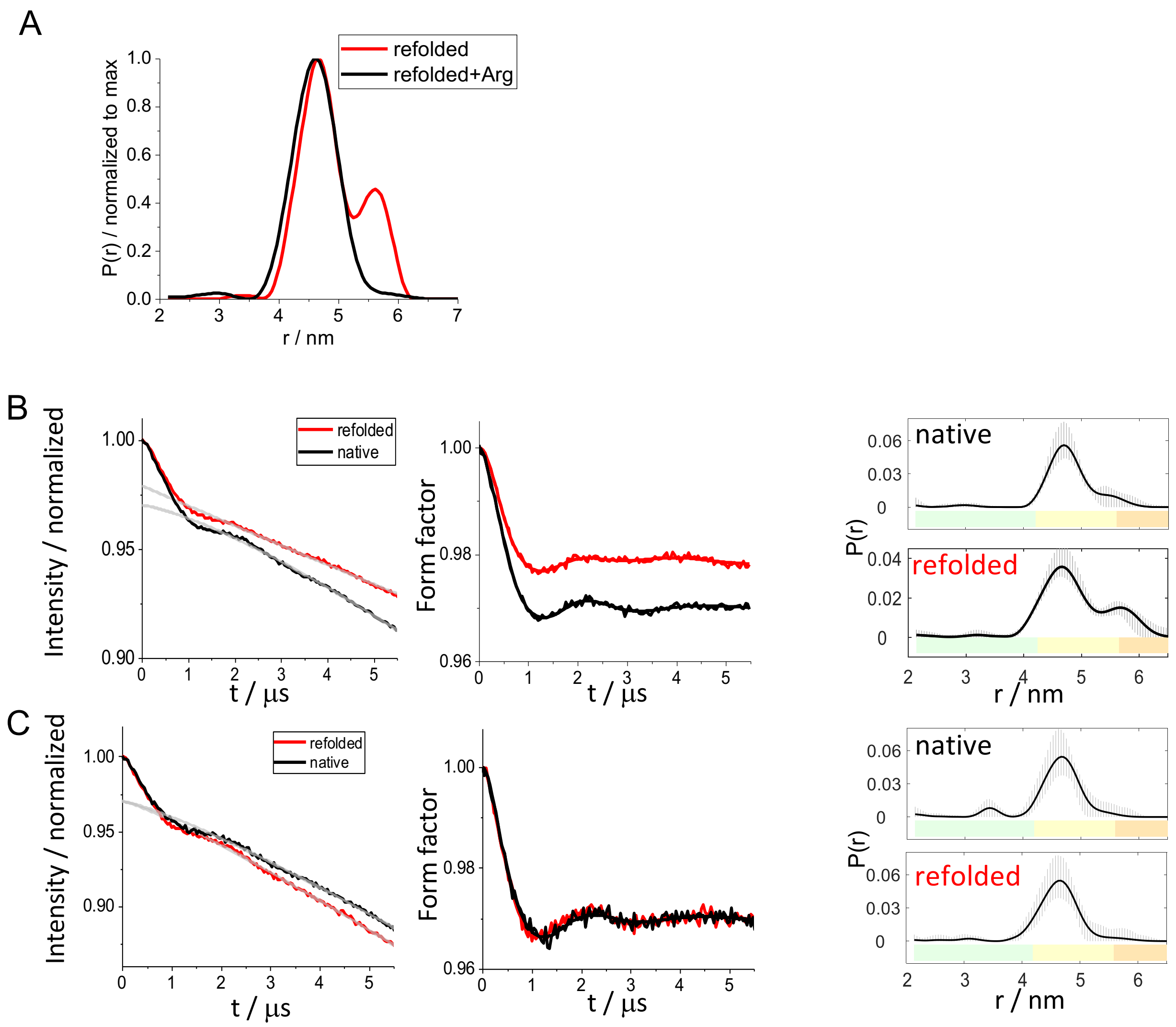

Supplementary Figure 6. DEER data for *Se*LAOBP samples. (A) Comparison of Gd(III)–Gd(III) distance distributions of refolded *Se*LAOBP tagged at positions 61 and 151, in the absence (red) and presence (black) of a 1.5 molar excess of L-arginine. (B-C) Raw and processed data from DEER measurements of natively purified (black) and refolded (red) *Se*LAOBP samples in the (B) absence and (C) presence of a 1.5 molar excess of L-arginine. Samples were measured using 100 µM protein in D_2O and 20 % w/v deuterated glycerol. The left panel shows the primary DEER data for the sample and background (gray) decay function. The central panel shows the DEER form factors after background correction, with fits corresponding to the distance distributions shown in the right-hand side panel. The right panel shows the corresponding distance distributions: the solid lines represent the distributions with the best r.m.s.d. from the experimental data and the striped regions represent the variation of alternative distributions (± 2 times the standard deviation) obtained by varying the parameters of the background correction and noise as calculated by the validation tool in the DeerAnalysis software package[5]. The parameter ranges used for the

validations were: white noise 0–1.5, background start $0.2*t_{max}$–$0.6*t_{max}$, and background dimension 3–3.6. The colored bars at the bottom of each panel reflect the reliability of each region of the distribution, according to DEER evolution time used, as defined in DeerAnalysis (pale green: the shape of the distance distribution is reliable; pale yellow: the mean distance and distribution width are reliable; pale orange: the mean distance is reliable; pale red: long-range distance contributions may be detectable, but cannot be quantified).

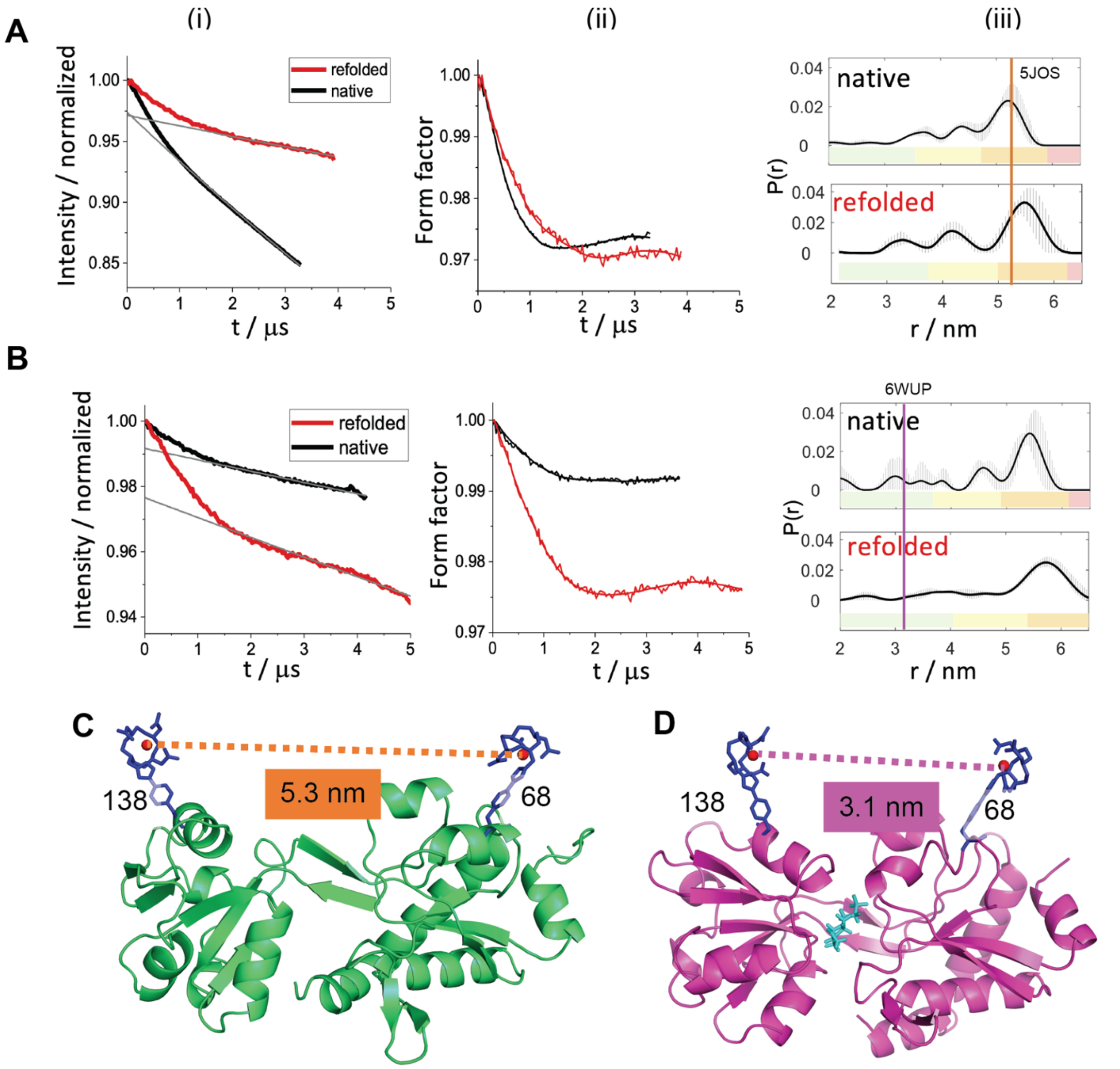

Supplementary Figure 7. Primary DEER data for AncCDT-3/P188 and AncCDT-5 samples. Primary data from the DEER measurements (as shown in Figure 3) on natively purified (black) and refolded (red) of (A) AncCDT-3/P188 tagged at positions 68 and 138, and (B) AncCDT-5 tagged at positions 68 and 138. Samples were measured using 100 µM protein in D_2O and 20 % w/v deuterated glycerol. The left panel (i) shows the primary DEER data for the sample and background (gray) decay function. The central panel (ii) shows the DEER form factors after background correction, with fits corresponding to the distance distributions shown in (iii). The right panel (iii) shows the corresponding distance distributions: the solid lines represent the distributions with the best r.m.s.d. from the experimental data and the striped regions represent the variation of alternative distributions (± 2 times the standard deviation) obtained by varying the parameters of the background correction and noise as calculated by the validation tool in the

DeerAnalysis software package[5]. The parameter ranges used for the validations were: white noise 0–1.5, background start 0.2*t_{max}–0.6*t_{max}, and background dimension 3–3.6. The colored bars at the bottom of each panel reflect the reliability of each region of the distribution, according to DEER evolution time used, as defined in DeerAnalysis (pale green: the shape of the distance distribution is reliable; pale yellow: the mean distance and distribution width are reliable; pale orange: the mean distance is reliable; pale red: long-range distance contributions may be detectable, but cannot be quantified). In (B), the primary DEER traces were shortened in the analysis to avoid an instrumental artefact. Vertical lines represent the predicted Gd(III)–Gd(III) distances when AzF-propargyl-DO3A-Gd(III) residues are modelled onto crystal structures of AncCDT-3/L188 (PDB 5JOS) at positions 68 and 138, or AncCDT-5 (PDB 6WUP) at positions 68 and 138. As the main peak in each DEER distance distribution appears in the orange region, we also calculated means of the distance distributions to assess potential contributions of the smaller peaks nearby. We considered two ranges for the calculation, where the first range (4.0–6.5 nm) included the small peak appearing in the yellow region next to the main peak while the second range included only the most intense peak (5.0–6.5 nm). The mean distance values obtained were: (A) native: 4.96 ± 0.42 and 5.18 ± 0.26 nm (original peak maximum at 5.2 nm); (A) refolded: 5.15 ± 0.57 and 5.44 ± 0.24 nm (original peak maximum at 5.4 nm); (B) native: 5.26 ± 0.41 nm and 5.47 ± 0.20 nm (original peak maximum at 5.4 nm); (B) refolded: 5.62 ± 0.24 (original peak maximum at 5.7 nm).

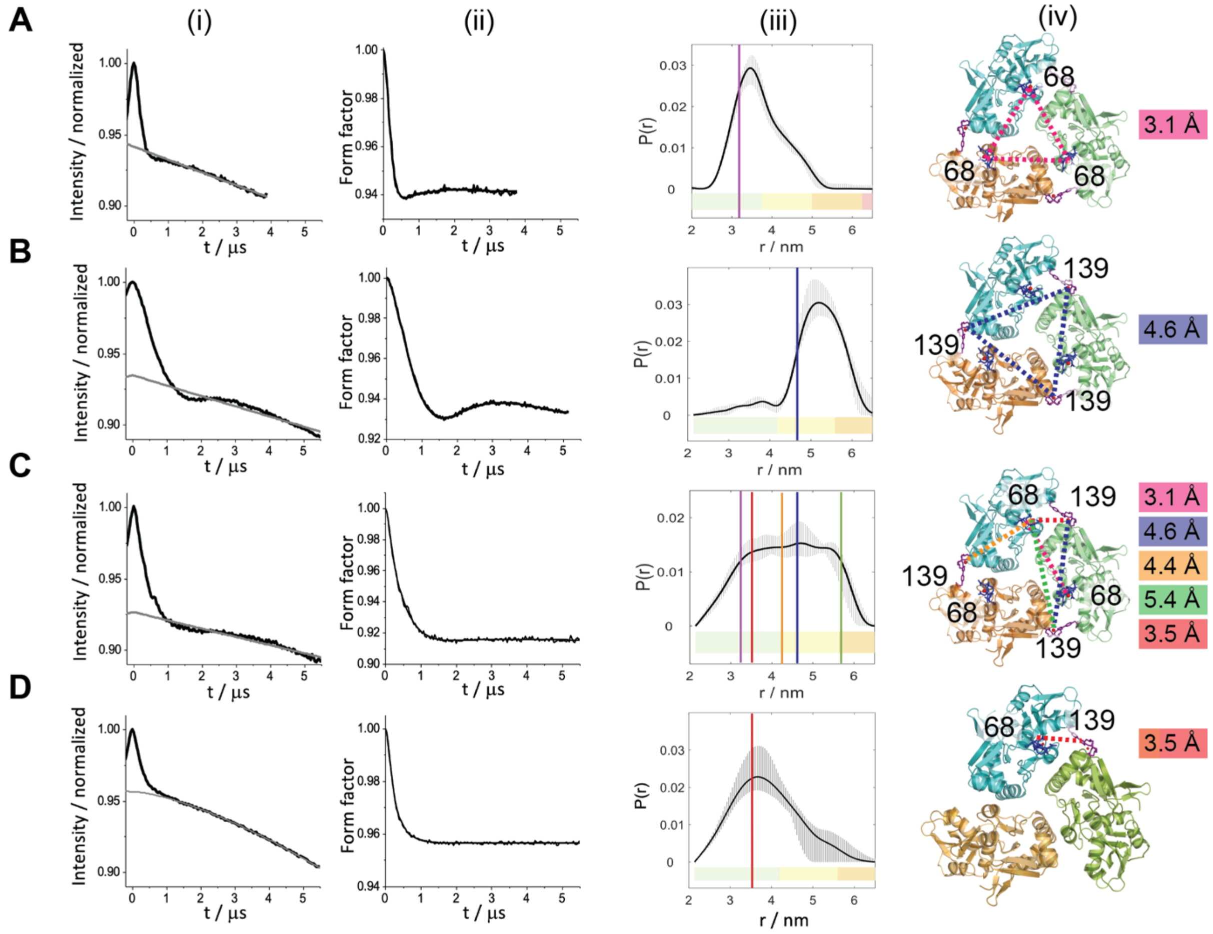

Supplementary Figure 8. Additional and primary DEER data for *Pa*CDT samples. Data from the DEER measurements on natively purified samples of *Pa*CDT tagged at (A) position 68, (B) position 139, (C) position 68 and 139, or (D) position 68 and 139, but following a 10-fold dilution with unlabelled *Pa*CDT for selective observation of the intramolecular distance between the two domains (as shown in Figure 3). Samples were measured using 100 µM protein in D_2O and 20 % w/v deuterated glycerol. The left panel (i) shows the primary DEER data for the sample and background (gray) decay function. The central panel (ii) shows the DEER form factors after background correction, with fits corresponding to the distance distributions shown in (iii). The right panel (iii) shows the corresponding distance distributions: the solid lines represent the distributions with the best r.m.s.d. from the experimental data and the striped regions represent the variation of alternative distributions (± 2 times the standard deviation) obtained by varying the parameters of the background correction and noise as calculated by the validation tool in the DeerAnalysis software package[5]. The parameter ranges used for the validations of (A)-(C) were: white noise 0–1.5, background start $0.2*t_{max}$–$0.6*t_{max}$, and background dimension 3–3.6. The parameter ranges used for the validation of (D) were the same, except that the background dimension was varied in the range of 3-4 to take into account the dimensionality of four we used

to get a good fit. The colored bars at the bottom of each panel reflect the reliability of each region of the distribution as defined in DeerAnalysis (pale green: the shape of the distance distribution is reliable; pale yellow: the mean distance and distribution width are reliable; pale orange: the mean distance is reliable; pale red: long-range distance contributions may be detectable, but cannot be quantified.). In (B), the primary DEER traces were shortened in the analysis to avoid an instrumental artefact. Vertical lines represent the predicted Gd(III)–Gd(III) distances when AzF-propargyl-DO3A-Gd(III) residues are modelled onto the crystal of structure *Pa*CDT (PDB 3KBR) at tagged positions, as shown in panel (iv).

```
AncCDT-1        1    AASTLDEIMKRGTLRVGTDADYKPFSFKDKNGQYTGFDIDLAKALAKELGVKVEFVPTTW
AncCDT-3/P188   1    AASRLDEIMERGTLRVGTTGDYKPFSYRDPDGQYTGFDIDVAKSLAKSLGVKVEFVPTTW
AncCDT-3/L188   1    AASRLDEIMERGTLRVGTTGDYKPFSYRDPDGQYTGFDIDVAKSLAKSLGVKVEFVPTTW
AncCDT-5        1    AASRLDEIMQRGTLRVGTTGDYKPFSYRDPDGQFTGFDIDMAESLAKSLGVKVEFVPTTW
PaCDT           1    QESRLDRILESGVLRVATTGDYKPFSYRTEEGGYAGFDVDMAQRLAESLGAKLVVVPTSW

AncCDT-1        61   DGIIPALQTGKFDIVMSGMTITPERKKKVDFSDPYMTAGQTILVKKDNADKIKSFEDLNK
AncCDT-3/P188   61   PTLMSDLQADKFDIAMGGVTVTPERQKKADFSDPYMTFGKTPLVRKEDADKFKSLEDINR
AncCDT-3/L188   61   PTLMSDLQADKFDIAMGGVTVTPERQKKADFSDPYMTFGKTPLVRKEDADKFKSLEDINR
AncCDT-5        61   PTLMDDFQADKFDIAMGGVSVTPERQKKADFSEPYMTDGKTPIVRCEDADKYQTLEQIDR
PaCDT           61   PNLMRDFADDRFDIAMSGISINLERQRQAYFSIPYLRDGKTPITLCSEEARFQTLEQIDQ

AncCDT-1        121  PDVKVAVQLGTTSEQAAKEFLPKAKIRTFENNAEAFQEVVSGRADAMVTDSPVAAYYAKK
AncCDT-3/P188   121  PDVRVAVNPGGTNEKFAREHLKKAKITVYENNVEIFQEVASGRADVMITDTVEALYYAKK
AncCDT-3/L188   121  PDVRVAVNPGGTNEKFAREHLKKAKITVYENNVEIFQEVASGRADVMITDTVEALYYAKK
AncCDT-5        121  PDVRVVVNPGGTNERFARAHLKQAQITVYPDNVTIFQEIVAGRADVMMTDAVETRYQQKL
PaCDT           121  PGVTAIVNPGGTNEKFARANLKKARILVHPDNVTIFQQIVDGKADLMMTDAIEARLQSRL

AncCDT-1        181  NPGLAVVVVDEPFTHEPLGFAIRKGDPELLNWVNNWLKQMKKDGTYDKLYEKWFK.....
AncCDT-3/P188   181  HPGLAAVPVDKPFTHSEKGYMMPKGDQEFLNYVNQWLDQMKQQGTYEKLYEKWLK.....
AncCDT-3/L188   181  HPGLAAVLVDKPFTHSEKGYMMPKGDQEFLNYVNQWLDQMKQQGTYEKLYEKWLK.....
AncCDT-5        181  HPGLCAVHVDKPFTHSEKAYLLPRGDPAFKAYVDQWLHQAMQSGTYQRIFDKWLK.....
PaCDT           181  HPELCAVHPQQPFDFAEKAYLLPRDEAFKRYVDQWLHIAEQSGLLRQRMEHWLEYRWPTA

AncCDT-1             ...
AncCDT-3/P188        ...
AncCDT-3/L188        ...
AncCDT-5             ...
PaCDT           241  HGK
```

Supplementary Figure 9. Multiple sequence alignment. Multiple sequence alignment of the proteins studied in this work. N-terminal residues that were introduced during cloning, including the His_6-tag, have been omitted. The residue numbering corresponds to that used throughout this work. The sequences were aligned using Clustal Omega[2]. The figure was generated using ESPript 3[3]. Every 10th residue position in the alignment is indicated by a black dot. Shading is based on the percentage of equivalent residues at that position (i.e. black = 100 % conserved).

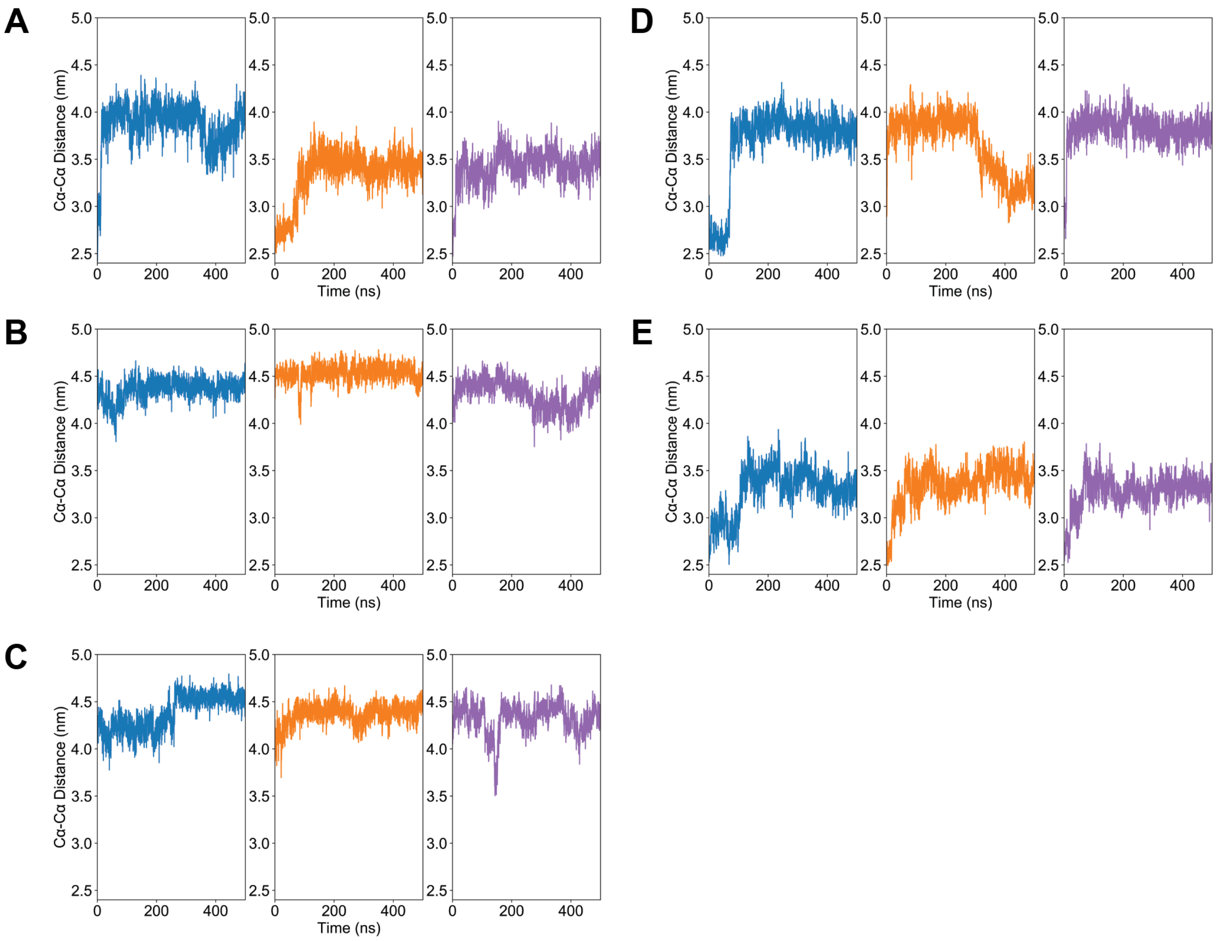

Supplementary Figure 10. Time evolution of Cα–Cα distances in MD simulations. The time evolution of distances between the alpha carbons of tagging sites in simulations of (A) AncCDT-1, (B) AncCDT-3/P188, (C) AncCDT-3/L188, (D) AncCDT-5 and (E) *Pa*CDT. For AncCDT-1, AncCDT-3 and AncCDT-5, individual replicates (n=3) are shown as different colored traces. For the *Pa*CDT plot, the Cα–Cα distances between the tagged sites of each monomer of the *Pa*CDT trimer (n=1) are shown as different colored traces.

Supplementary References

1. Mahawaththa, M. C. *et al.* Small neutral Gd(III) tags for distance measurements in proteins by double electron-electron resonance experiments. *Phys. Chem. Chem. Phys.* **20**, 23535–23545 (2018).
2. Sievers, F. *et al.* Fast, scalable generation of high-quality protein multiple sequence alignments using Clustal Omega. *Mol. Syst. Biol.* **7**, 539 (2011).
3. Robert, X. & Gouet, P. Deciphering key features in protein structures with the new ENDscript server. *Nucleic Acids Res.* **42**, W320–W324 (2014).
4. Abdelkader, E. H. *et al.* Protein conformation by EPR spectroscopy using gadolinium tags clicked to genetically encoded *p*-azido-L-phenylalanine. *Chem. Commun.* **51**, 15898–15901 (2015).
5. Jeschke, G. *et al.* DeerAnalysis2006—a comprehensive software package for analyzing pulsed ELDOR data. *Appl. Magn. Reson.* **30**, 473–498 (2006).
