# Supplementary material for: Altered conformational sampling along an evolutionary trajectory changes the catalytic activity of an enzyme
Source: Nat Commun. 2020 Nov 23;11:5945. doi: 10.1038/s41467-020-19695-9 (PMC7683729; doi:10.1038/s41467-020-19695-9)
Supplement: Supplementary file 3 — Reporting Summary [file 41467_2020_19695_MOESM3_ESM.pdf]

nature research

Corresponding author(s): Colin J Jackson

Last updated by author(s): Oct 8, 2020

Reporting Summary

Nature Research wishes to improve the reproducibility of the work that we publish. This form provides structure for consistency and transparency in reporting. For further information on Nature Research policies, see Authors & Referees and the Editorial Policy Checklist .

Statistics

For all statistical analyses, confirm that the following items are present in the figure legend, table legend, main text, or Methods section.

n/a	Confirmed	
☐	☒	The exact sample size (*n*) for each experimental group/condition, given as a discrete number and unit of measurement
☐	☒	A statement on whether measurements were taken from distinct samples or whether the same sample was measured repeatedly
☒	☐	The statistical test(s) used AND whether they are one- or two-sided<br>*Only common tests should be described solely by name; describe more complex techniques in the Methods section.*
☒	☐	A description of all covariates tested
☒	☐	A description of any assumptions or corrections, such as tests of normality and adjustment for multiple comparisons
☐	☒	A full description of the statistical parameters including central tendency (e.g. means) or other basic estimates (e.g. regression coefficient) AND variation (e.g. standard deviation) or associated estimates of uncertainty (e.g. confidence intervals)
☒	☐	For null hypothesis testing, the test statistic (e.g. *F*, *t*, *r*) with confidence intervals, effect sizes, degrees of freedom and *P* value noted<br>*Give P values as exact values whenever suitable.*
☒	☐	For Bayesian analysis, information on the choice of priors and Markov chain Monte Carlo settings
☒	☐	For hierarchical and complex designs, identification of the appropriate level for tests and full reporting of outcomes
☒	☐	Estimates of effect sizes (e.g. Cohen's *d*, Pearson's *r*), indicating how they were calculated

Our web collection on statistics for biologists contains articles on many of the points above.

Software and code

Policy information about availability of computer code

Data collection	Maestro (Schrödinger Release 2019-1: Maestro, Schrödinger, LLC, New York, NY, 2019) was used for MD simulations.
Data analysis	Several common software was used for this project:<br>GraphPad Prism 8 (8.3.1) was used to produce figures and plots. The PyMOL Molecular Graphics System (ver 2.3.2) was used to produce figures of protein structures. DeerAnalysis (2006) was used for analysis of DEER data. ProDy (v1.10.10) was used for analysis of MD simulations. ASTRA (ver 5.3.4.) was used for SEC-MALS analysis. ESPcript (3.0) was used for producing the multiple sequence alignment figure.<br>For crystallography:<br>XDS (ver 2019) was used for data indexing and integration. Aimless (0.7.3) was used for scaling. Phaser (2.7.0) was used for molecular replacement. Coot (0.8.9.2), phenix.refine (Phenix 1.17.1-3660) and Refmac (5.0.32) were used for refinement.

For manuscripts utilizing custom algorithms or software that are central to the research but not yet described in published literature, software must be made available to editors/reviewers. We strongly encourage code deposition in a community repository (e.g. GitHub). See the Nature Research guidelines for submitting code & software for further information.

Data

Policy information about availability of data

All manuscripts must include a data availability statement. This statement should provide the following information, where applicable:

- Accession codes, unique identifiers, or web links for publicly available datasets
- A list of figures that have associated raw data
- A description of any restrictions on data availability

The atomic coordinates and structure factors for AncCDT-5 have been deposited in the Worldwide Protein Data Bank (PDB 6WUP). Raw data underlying Supplementary Figure 1 are supplied in Source Data file. All other raw data are available upon request.

Field-specific reporting

Please select the one below that is the best fit for your research. If you are not sure, read the appropriate sections before making your selection.

☒ Life sciences ☐ Behavioural & social sciences ☐ Ecological, evolutionary & environmental sciences

For a reference copy of the document with all sections, see nature.com/documents/nr-reporting-summary-flat.pdf

Life sciences study design

All studies must disclose on these points even when the disclosure is negative.

Sample size	Sample sizes were chosen based on accepted methods in the field.
Data exclusions	No data was excluded.
Replication	Three technical repeats were performed for prephenate dehydratase activity determination with consistent results. MD simulations were performed in triplicate (except for PaCDT), with consistent results. A single MD simulation of PaCDT was performed, since the dynamics of the three individual chains in the trimer could be assessed separately. Other measurements were obtained from single experiments as is common for these techniques.
Randomization	N/A. No randomization was required for this study, as is normal in this field.
Blinding	N/A. Blinding was not performed for this study, as is normal in this field.

Reporting for specific materials, systems and methods

We require information from authors about some types of materials, experimental systems and methods used in many studies. Here, indicate whether each material, system or method listed is relevant to your study. If you are not sure if a list item applies to your research, read the appropriate section before selecting a response.

Materials & experimental systems

n/a	Involved in the study	
☒	☐	Antibodies
☒	☐	Eukaryotic cell lines
☒	☐	Palaeontology
☒	☐	Animals and other organisms
☒	☐	Human research participants
☒	☐	Clinical data

Methods

n/a	Involved in the study	
☒	☐	ChIP-seq
☒	☐	Flow cytometry
☒	☐	MRI-based neuroimaging
